# The prokineticin system and glia cells as pharmacological targets to control neuroinflammation and to relieve pain in a murine model of Fabry–Anderson disease

**DOI:** 10.1097/j.pain.0000000000003818

**Published:** 2025-09-25

**Authors:** Giulia Galimberti, Silvia Franchi, Giada Amodeo, Patrizia Romualdi, Sanzio Candeletti, Laura Rullo, Loredana Maria Losapio, Valentina Onnis, Davide Moi, Benedetta Riboldi, Giulia Magni, Stefania Ceruti, Paola Sacerdote

**Affiliations:** a Department of Pharmacological and Biomolecular Sciences “Rodolfo Paoletti”, Università degli Studi di Milano, Milan, Italy; b Department of Pharmacy and Biotechnology, Alma Mater Studiorum-University of Bologna, Bologna, Italy; c Department of Life and Environmental Sciences, University of Cagliari, Cagliari, Italy

**Keywords:** Neuroinflammation, Pain, Prokineticin system, Glial cells, Minocycline, Fabry disease, *GLA*^−/−^ mice

## Abstract

Neuropathic pain is a major symptom of Fabry–Anderson disease (FD). It develops in childhood, is life-lasting, and resists current therapies; finding new therapeutic strategies is urgently needed. We demonstrate that neuroinflammation control effectively relieves FD pain. We used 2 pharmacological approaches: the microglial inhibitor minocycline and the block of the activity of the chemokine prokineticin-2 with the specific receptor antagonist PC1 (patented compound 1). Ten- and 25-week-old male *GLA* ^*−/−*^ mice (the FD murine model) were used. These mice were characterized by mechanical allodynia, thermal hyperalgesia, hyposensitivity to cold stimuli, and abdominal pain. Two weeks of treatment with minocycline or PC1 successfully counteracted sensory alterations. A significant inflammatory state, characterized by high levels of prokineticin-2 and proinflammatory cytokines, was present in the FD gut. The sciatic nerve showed initial severe neuroinflammation that attenuated over time. In dorsal root ganglia, neuroinflammation was severe and persistent with prokineticin-2, proinflammatory cytokines, ionized calcium-binding adapter molecule 1, and glial fibrillary acidic protein overexpression; histone demethylases KDM6A and B were also upregulated. We highlighted neuroinflammation in the spinal cord that increased over time. Treatment with minocycline or PC1 significantly counteracted inflammation and neuroinflammation, reducing prokineticin-2 and proinflammatory cytokines levels and increasing anti-inflammatory factor PPARγ expression. Both treatments prevented the onset of micro- and astrogliosis in the spinal cord. We underline the role of neuroinflammation and microglia in FD pain and suggest that treatments that control the activity of the prokineticin system, glial activation, and the production of proinflammatory cytokines and increase anti-inflammatory mediators have a therapeutic effect on pain in FD.

## Abstract 2

Supplemental Digital Content is Available in the Text.

Neuropathic pain is the major untreated condition of Fabry–Anderson disease. Prokineticin-2 antagonism and the block of neuroinflammation are a winning strategy to control it.

## 1. Introduction

Fabry–Anderson disease (FD) is a hereditary lysosomal storage disorder caused by a deficiency of the α-galactosidase A (α-Gal A) enzyme, encoded by the *GLA* gene on the X chromosome. This deficiency leads to the accumulation of globotriaosylceramide (Gb3) and its metabolites in lysosomes. Fabry–Anderson disease prevalence ranges from 1:40,000 to 1:117,000, although newborn screening suggests it may be as frequent as 1:1400.^13^ It affects both men and women, but because of its X-linked inheritance, hemizygous men are more often and severely affected.^40^

Because α-Gal A is ubiquitous, FD is a multisystem disorder. One of the earliest and most prevalent symptoms is neuropathic pain (NP), which affects more than 59% of male and 41% of female patients,^17^ beginning between the ages of 2 and 4 years.^40^ Gb3 accumulation in the nervous system leads to sensory nerve damage and dorsal root ganglia (DRG) impairment, resulting in chronic NP.^17^ This symptom severely affects patients' lifestyle and psychological state,^2,23^ persisting into adulthood in more than 80% of cases.^7,13,33,52,57,58,62^ Current therapies, such as enzyme replacement therapy (ERT) and the chaperone migalastat, have limited impact on NP.^25,52,57,58,61^ Relief is typically obtained only with traditional analgesics (Non steroidal antinflammatory drugs, opioids, gabapentinoids), which often have side effects and limited efficacy.^52^

Therefore, identifying new and safer therapeutic targets for NP in FD is crucial. Neuropathic pain pathogenesis involves neuronal dysfunction and neuroinflammation, contributing to the sensitization of sensory neurons.^16,54,56^ In particular, in FD, microglia are activated by neuronal stress or Gb3 accumulation, undergo morphological changes, and release proinflammatory cytokines (eg, tumor necrosis factor-α [TNF-α], interleukin [IL-1β], IL-6), chemokines, and reactive oxygen species.^59^ Moreover, lipid accumulation may disturb neuron–glia interactions, exacerbating pain. Consistently, several animal models of NP show microglial activation and spinal cord neuroinflammation associated with pain behaviors.^21,37,41,42,67^

Targeting neuroinflammation could, therefore, be a promising strategy for managing NP in FD. In our study, we used the most validated murine model of FD (GLA^−/−^ mouse) at both early and advanced stages of the disease.^13,27^ We tested 2 pharmacological interventions: minocycline, a microglial inhibitor, and PC1 (patented compound 1), a specific antagonist of the chemokine prokineticin-2 (PK2).

We identified PK2 as a proinflammatory and pronociceptive mediator. Together with its prokineticin receptors PKR1 and PKR2, it forms the prokineticin system (PKS), expressed in immune cells, glia, and neurons in pain-related areas as the sciatic nerve, DRG, and spinal cord.^4–6,19–21,37,41–46,64^ The PKS is involved in neuroinflammation and chronic pain,^4–6,15,21,37,41,42^ and its inhibition with PC1 reduces pain symptoms by dampening inflammation and modulating epigenetic mechanisms.^6,15,21,41,42,49,51,53^

Minocycline, a semisynthetic antibiotic, is emerging as a treatment for conditions involving microglial activation,^22,68^ with analgesic effects in several preclinical pain models.

Our approaches, targeting microglial activation and the PK2 pathway, may represent effective therapeutic opportunities for alleviating chronic pain in FD.

## 2. Materials and methods

### 2.1. Animals

All experiments were performed on both young (10-week-old, n = 24) and adult (25-week-old, n = 24) male mice knockout for the *GLA* gene, encoding for α-galactosidase A, (GLA^*−/−*^ mice*;* FD; strain #003535) and wildtype (B6129SF2/J; Control [CTR]; strain #101045) mice (The Jackson Laboratory, Bar Harbor, ME).^13,27,47^ Animals were housed in 3 per cage (Macrolon type II cages—26 cm × 20 cm × 14 cm), each equipped with chipboard bedding, nesting materials, and environmental enrichment. They were maintained in standard environmental conditions (12 hours light/dark cycle at 22 ± 1°C and humidity of 55 ± 10%) and fed with dry pellets and water ad libitum. Periodic veterinary checks were performed during the entire experimental protocol period. All mice were acclimatized for 2 weeks before starting the procedures which were conducted following the guidelines defined by the Animal Care and Use Committee of the Italian Ministry of Health (DLGs 26/2014; Authorization 171/2023 to P.S.) and the European Community directives which regulate animal research (2010/63/UE). The 3 Rs (Replacement, Reduction, Refinement) principle was adopted and respected, guaranteeing the model as the only one useful for our research purposes, minimizing the number of animals used and their suffering.

### 2.2. Pharmacological treatments

After assessing basal thresholds, control and FD mice of both age groups were randomized into 4 experimental groups (CTR, FD, FD + PC1, and FD + minocycline; n = 6 per group) using a computerized randomization method. The 14-day treatment period (from day 0 to day 14) with either drugs or vehicle was then initiated. Fabry–Anderson disease mice were treated with the nonpeptidic antagonist of the PKS PC1 (150 μg/kg, subcutaneously [s.c.], twice a day),^8,15,21,41,42^ with the inhibitor of microglia minocycline (10 mg/kg, intraperitoneally [i.p.], once a day, Sigma-Aldrich)^1^ or with vehicle (saline solution, 10 μL/g). CTR mice (wildtype B6129SF2/J) were treated with vehicle as well. Considering that the compounds were administered by 2 different routes, we decided to give the vehicle to half of these animals i.p. and half s.c. Because no behavioral or biochemical differences were observed concerning the route of administration, data obtained have been pooled for statistical analysis. All drugs were freshly prepared, and all the animals were killed after the last drug administration (day 14).

### 2.3. Behavioral tests

The pain-like responses in 10- and 25-week-old FD mice were evaluated and compared with the age-matched healthy CTR mice to verify that this preclinical model well-recapitulated the sensory alterations reported by patients with FD. Mechanical allodynia (von Frey test), thermal hyperalgesia (plantar test), referred abdominal pain (von Frey filament test), and sensitivity to cold stimuli (cold plate test) were tested.

In both 10- and 25-week-old mice, thermal hyperalgesia and mechanical allodynia were tested before (0, basal) and after 4, 7, and 14 days of chronic pharmacological treatments, while referred abdominal pain and sensitivity to cold stimuli were evaluated at the end of treatments (day 14). Moreover, the acute antiallodynic effect of both PC1 and minocycline was also assessed after 30, 60, 90, and 120 minutes from the first drug injection in all animals.

Behavioural tests were performed in the morning, between 8 and 10 am, before daily drug administrations and 12 to 14 hours from the previous injections.

All the evaluations were executed after 30 minutes of animals' acclimatization to the test environment by trained researchers blinded to genotype and treatments.

#### 2.3.1. Mechanical allodynia: von Frey test

The von Frey test, with the dynamic plantar aesthesiometer (Ugo Basile, Gemonio, Italy), was used to evaluate mechanical allodynia. Mice were placed in a plexiglass cage (w 8.5 cm × h 8.5 cm) over a metallic grid and the palm area between the plantar pads of their hind paws was mechanically stimulated using the von Frey filament (metal filament, 0.5 mm diameter). The test started with a force value below the detection threshold and continued with an increased force, ranging up to 10 g in 10 seconds until the animal removed its paw or reached the cut-off (10 g).^3,15,21,41^ The test was conducted in both right and left paws (2 measurements/paw), and the response to mechanical stimuli (paw withdrawal threshold) expressed in grams (g) was recorded and averaged.

#### 2.3.2. Thermal hyperalgesia: plantar test

Thermal hyperalgesia was assessed based on the Hargreaves procedure, slightly modified by us,^3^ using a plantar test apparatus (Ugo Basile). Each mouse was located in a plexiglass box (w 11 × h 11 cm) and the palm area between the plantar pads of their hind paws was stimulated through a constant radiant intensity heat beam (Ø 0.5 cm, Ω 20). The seconds (cut-off 22 seconds) needed to withdraw the paw were recorded (paw withdrawal latency). The test was conducted on both the hind paws of each animal, and the right and left paw's responses (2 measurements/paw) were averaged.

#### 2.3.3. Referred abdominal pain: von Frey filament test

Referred abdominal mechanical allodynia was assessed using calibrated von Frey filament (0.02, 0.04, 0.07, 0.16, 0.4, 0.6, 1, 1.4, 2 g—Ugo Basile). The test started with a medium filament (0.4 g), smoothly glided from each mouse's pelvic area to the diaphragm and back for 3 seconds. If the mouse positively reacted to the stimulation, a one-size smaller filament was tested; if no response was observed, the test continued with a one-size larger filament. Positive responses to the mechanical stimulation were abdominal retractions, licking or scratching of the stimulation area, and jumping. The 50% withdrawal threshold was determined according to the simplified up-down method and calculated by Up-Down Reader (v2.0).^5,24,50^

#### 2.3.4. Sensitivity to cold stimuli: cold plate test

The cold plate test (Ugo Basile) was used to evaluate sensitivity to cold stimuli. The animals were placed in a plexiglass cylinder over the plate, previously brought to a temperature of 0°C. During the test, the animal behavior was observed and in the presence of any pain-like reaction, such as a jump, a tremor, and a flicking/licking of the front or hind legs, the test was concluded, and the reaction time (s) was recorded. In the absence of any pain-like response, the test was concluded at a cut-off of 60 seconds to preserve tissue integrity.^60^

### 2.4. Tissue collection

At the end of the experimental protocol, ie, after 14 days of treatment with PC1 or minocycline, mice of both ages were killed by decapitation. The colon-rectum tissues, sciatic nerves, DRG (L4-L6, and spinal cord (L4-L6) were isolated and collected according to standardized laboratory procedures.^3,5,21^ All tissues were frozen in liquid nitrogen and stored at −80°C until biochemical evaluations. The tissue for both mRNA and protein analyses was obtained from the same animal: All the tissues were divided into equal parts, separating the right from the left sides.

### 2.5. Real time quantitative polymerase chain reaction (RT-qPCR)

RNA was extracted using the TRIzol reagent (Invitrogen, Waltham, MA) according to the manufacturer's instructions. In brief, after adding TRIzol, tissues were homogenized using Ultra Turrax (T25 Janke & Kunkel- IKA Labor Technik, Staufen, Germany) for gut and an ultrasonic processor (UP50H DR. Hielscher, Teltow, Germany) for all the other tissues (sciatic nerve, DRG, and spinal cord). From the extracted RNA, cDNA was obtained thanks to the reverse transcriptase kit LunaScript (BioLabs, London, United Kingdom) and used as a template in quantitative PCR (QuantStudio5). Real-time PCR was executed using Luna Universal Probe, qPCR Master Mix (BioLabs), and specific Taqman Gene expression assays (Thermofisher Scientific, Waltham, MA) as prokineticin 2 (PK2: Mm01182450_g1), prokineticin receptors (PKR1: Mm00517546_m1 and PKR2: Mm00769571_m1), interleukin 6 (IL-6: Mm00446190_m1), interleukin-1β (IL-1β: Mm00434228_m1), tumor necrosis factor-α (TNF-α: Mm00443258_m1), glial fibrillary acidic protein (GFAP: Mm01253033_m1), ionized calcium-binding adapter molecule 1 (Iba1: Mm00479862_g1), and Glyceraldehyde-3-phosphate dehydrogenase (GAPDH:Mm99999915_g1). Conversely, to analyze PPARγ, KDM6A, and KDM6B gene expression, the SYBR Green PCR MasterMix was used (Life Technologies, Carlsbad, CA). The primers used for PCR amplification in SYBR Green PCR MasterMix were as follows:

GAPDH Forward 5′-AAC​TTT​GGC​ATT​GTG​GAA​GG-3′;

GAPDH Reverse 5′-ACA​CAT​TGG​GGG​TAG​GAA​CA-3′;

KDM6A Forward 5′-TTT​GGT​CTA​CTT​CCA​TTA​CAA​TGC​A-3′;

KDM6A Reverse 5′-AAG​CCC​AAG​TCG​TAA​ATG​AAT​TTC-3′;

KDM6B Forward 5′-ACC​GCC​TGC​GTG​CCT​TAC-3′;

KDM6B Reverse 5′-GTG​TTG​CTG​CTG​CTG​CTA​CT-3′;

PPARγ Forward 5′-GGA​AGA​CCA​CTC​GCA​TTC​CTT-3′;

PPARγ Reverse 5′-GTA​ATC​AGC​AAC​CAT​TGG​GTC​A-3′.

The mRNA levels of each gene were normalized to GAPDH and expressed as fold over the respective age-matched CTR levels. The data were analyzed using the 2^−∆∆Ct^ method. Each sample was run in triplicate.

### 2.6. Western blotting

Proteins were extracted according to the RIPA Lysis and Extraction Buffer's instructions (Thermo Fisher Scientific, Monza, Italy); the gut was homogenized using Ultra Turrax and the spinal cord using an ultrasonic processor (see above). Proteins were quantified by the Bradford method, and 40 μg of total proteins were mixed with Laemmli buffer, heated at 95°C for 9 minutes and loaded into a 10% (spinal cord proteins) or 15% (gut proteins) polyacrylamide gel.

The following primary antibodies were used: rabbit anti-PK2 (1:500; Cell Signaling Technology, Danvers, MA, #87360), rabbit anti-GFAP (1:1000; Cell Signaling Technology, #12389), and rabbit antiactin (1:1000; Cell Signaling Technology, #8457). Antirabbit-HRP (1:3000; Cell Signaling Technology, #7074) was used as a secondary antibody. Densitometric analysis of protein bands was performed thanks to ImageJ software. The expression of the protein of interest was normalized to the housekeeping (β-actin) protein expression and expressed as fold over the age-matched CTR protein levels.

### 2.7. Immunohistochemistry

Two FD and 2 CTR mice of both ages were used for the immunohistochemistry analyses. Mice were deeply anesthetized (60 mg/kg of sodium pentobarbital, i.p.) and intracardially perfused with phosphate buffered saline (PBS) followed by 4% formalin. After killing, the colon-rectal portion of the gut was isolated, opened longitudinally, cleaned, and rolled with the mucosa outward. Dorsal root ganglia and the spinal cord were excised. All tissues were postfixed in 10% formalin for 60 minutes, cryoprotected in 30% sucrose (48 hours), and embedded in optimal cutting temperature mounting medium (VWR, Milan, Italy) to be cut on a cryostat. The gut and spinal cord were coronally cut at 20 μm thickness sections, while DRG were cut at 10 μm. Sections were incubated for 45 minutes in PBS containing 10% normal goat serum (Life Technologies, Monza, Italy) and 0.3% Triton X-100 (Sigma-Aldrich, Merck group, Milan, Italy), and then overnight at 4°C with the following primary antibodies: rabbit anti-Prokineticin 2 (anti-PK2, 1:100; CovaLab, Bron, France, pab0409-P) or rabbit antiastrocyte marker GFAP (anti-GFAP, 1:600; Dako, Milan, Italy, Z0034). Sections were then rinsed 3 times with PBS and incubated for 1 hour RT with AlexaFluor 488-conjugated goat antirabbit secondary antibody (1:600; Life Technologies, Monza, Italy, A11008). All antibodies were diluted in PBS containing 0.1% Triton X-100 and 5% normal goat serum. Cell nuclei were counterstained with the Hoechst33258 dye (1:20,000; Sigma-Aldrich, Merck, 94003). Immunostaining negative controls were performed on all the analyzed tissues by omitting the primary antibodies (ie, anti-PK2 and anti-GFAP) and incubating with only the secondary antibody (AlexaFluor 488-conjugated goat antirabbit). Samples were examined with a laser scanning confocal microscope (LSM 510, Zeiss, Milan, Italy), and images were acquired and processed using the LSM Image Browser software (Zeiss).

### 2.8. Statistical analysis

Behavioral data are reported as the mean ± SEM of 6 mice/group; biochemical data are reported as the mean ± SD of 3 to 6 mice/group (see figure legends).

The variability in sample size is attributable to the exclusion of a limited number of data points identified as outliers by the Grubbs' test, as well as occasional technical issues during the extraction procedures. All exclusions were applied before statistical analysis.

Both mechanical allodynia and thermal hyperalgesia data were analyzed through the 2-way ANOVA, followed by the Tukey post hoc test. One-way ANOVA, followed by the Tukey post hoc test, was used for the evaluation of painful symptoms referred to both abdominal pain and sensitivity to cold stimuli, measured at the end of the drug treatments. Biochemical data were analyzed with the one-way ANOVA test, followed by the Šidák post hoc test. Statistical analysis was performed using GraphPad Prism 10 (San Diego, CA), and all analyses were considered statistically different with *P* ≤ 0.05. Pearson correlation analysis assessed the associations between the 2^−ΔΔCt^ of each investigated gene in the DRG of young and adult mice. The regular correlation was computed using the rcorr function from the Hmisc package in R (10.32614/CRAN.package.Hmisc; https://rpubs.com/minhtri/968611). rcorr computes a matrix of Pearson r-rank correlation coefficients for all possible pairs of genes in the matrix. The level of significance was set at *P* < 0.05.

## 3. Results

### 3.1. PC1 and minocycline treatments counteract sensory alterations in Fabry–Anderson disease mice

**Figure 1.**
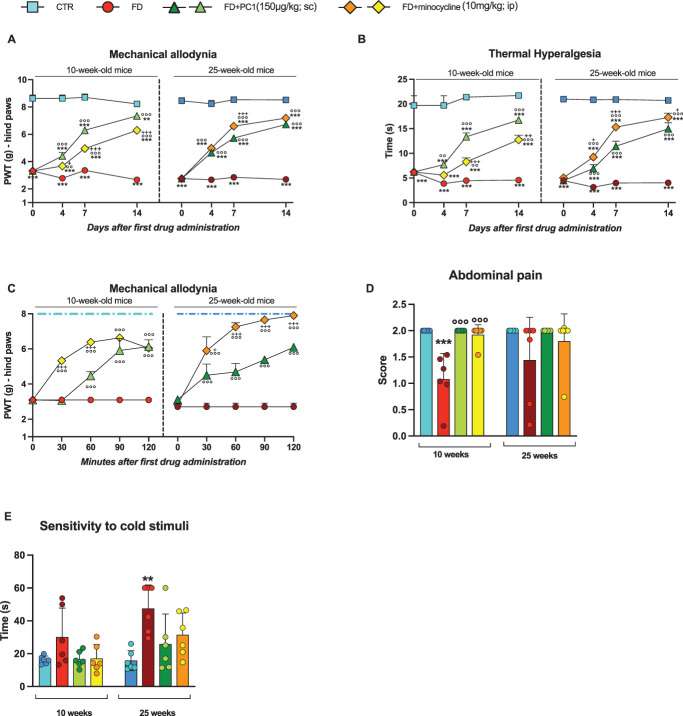
PC1 and minocycline treatments counteract sensory alterations in FD mice. The presence of sensory alterations was evaluated in both young (10-week-old) and adult (25-week-old) mice of all experimental groups. PC1 (150 µg/kg, s.c., twice a day) and minocycline (10 mg/kg, i.p., once daily) treatments were administered for 2 weeks. Basal evaluation (day 0) was performed before the beginning of the drug administration. (A) Mechanical allodynia was assessed using the von Frey test at days 0, 4, 7, and 14 from the beginning of the pharmacological treatments. (B) Thermal hyperalgesia was evaluated by the plantar test at days 0, 4, 7, and 14 from the first drug administration. (C) Mechanical allodynia was also estimated acutely after the first PC1 and minocycline administration (day 0) every 30 minutes up to 120 minutes. The dotted line refers to CTR values. (D) Abdominal pain (von Frey filament test) and (E) sensitivity to cold stimuli (cold plate test) were assessed at the end of the experimental protocol (day 14). Results are expressed as mean ± SEM of 6 animals/group. Statistical analysis was performed by 2-way ANOVA (A–C) or 1-way ANOVA (D–E) followed by the Tukey post hoc test. **P < 0.01, ***P < 0.001 vs age-matched CTR; ◦◦*P* < 0.01, ◦◦◦*P* < 0.001 vs age-matched FD; +*P* < 0.05, ++*P*< 0.01, +++*P* < 0.001 vs age-matched FD + PC1. Treatments × time: (A) 10 weeks: F (9,116) = 40.82, *P* < 0.0001; 25 weeks: F (9,116) = 71.87, *P* < 0.0001 (B) 10 weeks: F (9,116) = 15.42, *P* < 0.0001; 25 weeks: F (9,116) = 50.46, *P* < 0.0001(C) 10 weeks: F (8,75) = 16.25, *P* < 0.0001; 25 weeks: F (8,75) = 10.31, *P* < 0.0001 (D) 10 weeks: F (3,20) = 17.57 *P* < 0.0001; 25 weeks: F (3,20) = 1.795, *P* = 0.1807 (E) 10 weeks: F (3,20) = 2.639, *P* = 0.00775; 25 weeks: F (3,20) = 5.626, *P* = 0.0001. ANOVA, analysis of variance; FD, Fabry–Anderson disease.

Before initiating pharmacological treatments (day 0), a baseline evaluation of painful symptoms was performed in both young and adult GLA^−/−^ (FD) mice. As reported in Figure 1, all pathological animals exhibited a clear painful phenotype, characterized by significantly reduced response thresholds compared with their age-matched controls (CTR) after mechanical (panel A) and thermal (panel B) stimulation, indicating mechanical allodynia and thermal hyperalgesia (*P* < 0.001).

Subsequently, pharmacological treatments with the prokineticin system antagonist PC1 and the microglial inhibitor minocycline were performed for 2 weeks. The chronic effect of the 2 drugs was constantly evaluated in mice of both ages (panels A-B). In both young and adult mice, both compounds counteracted mechanical allodynia (panel A) and thermal hyperalgesia (panel B), significantly raising the response thresholds to the respective stimuli (*P* < 0.001 vs FD mice, day 14) over time, however, without managing to bring them back to the control level (*P* < 0.01 vs CTR mice).

Moreover, the acute antiallodynic effect of the drugs was also evaluated after the first administration in both young and adult mice (panel C). In the 10-week-old FD mice, minocycline significantly counteracted mechanical allodynia already 30 minutes after administration (*P* < 0.001 vs FD mice; *P* < 0.001 vs PC1 mice), while PC1 was effective starting from 60 minutes (*P* < 0.001 vs FD mice). However, at the last acute observation point (120 minutes), the antinociceptive effect of treatments was comparable. In the 25-week-old FD mice, both compounds had a similar antiallodynic trend with a very rapid positive effect (30 minutes from treatment, *P* < 0.001 vs FD mice) and with an overall greater effect of minocycline than PC1 (*P*< 0.05).

Because abdominal pain is a frequently reported symptom in patients with FD, the von Frey filament test was used to assess referred abdominal pain in young and adult FD mice at the end of the experimental protocol (day 14). Panel D clearly shows that 10-week-old FD mice presented referred abdominal pain (*P* < 0.001) compared with age-matched controls. Two weeks of either PC1 or minocycline administration significantly reduced the abdominal pain experienced by young pathological mice, restoring control conditions (*P* < 0.001 vs FD mice). By contrast, 25-week-old FD mice did not manifest significant differences in abdominal hypersensitivity in comparison with their age-matched controls. As expected, no significant effect of the drugs was observed.

We also checked the presence of sensitivity alterations in response to a cold stimulus in all mice (panel E) at the end of the treatment period (day 14). In comparison with the relative aged-control mice, in an earlier phase of the disease, pathological mice started to show a trend of hyposensitivity to cold (*P* = 0.1729), which became much more evident and statistically significant in 25-week-old FD mice (*P* < 0.01). The 2 pharmacological treatments ameliorated the symptoms, considering that no significant difference was observed between treated FD mice and age-matched controls.

### 3.2. Colon-rectum: prokineticin system and inflammatory markers and effect of PC1 and minocycline treatments

**Figure 2.**
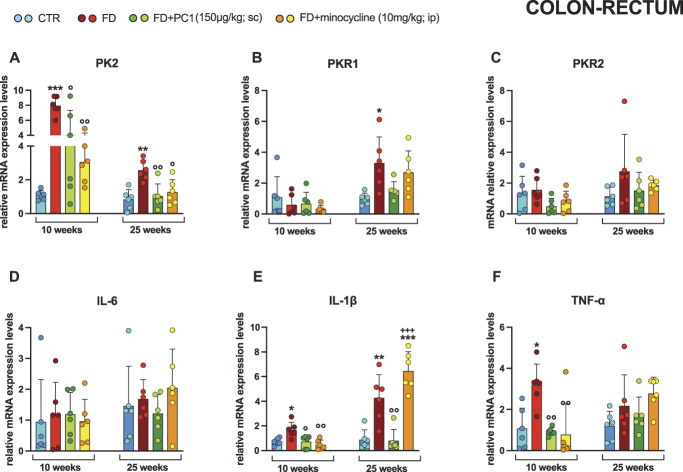
Colon-rectum: mRNA levels of PKS and inflammatory markers and effects of PC1 and minocycline treatments. mRNA expression levels (RT-qPCR) of the PKS members (A) PK2, (B) PKR1 and (C) PKR2, proinflammatory cytokines (D) IL-6, (E) IL-1β, and (F) TNF-α were evaluated in the colon-rectum (after 14 days of pharmacological treatment). Results are normalized to the housekeeping gene GAPDH and expressed as fold over the age-matched CTR group. Data are the mean ± SD of 6 animals/group. Statistical analyses were performed by 1-way ANOVA followed by the Šidák post hoc test. *P < 0.05, **P < 0.01, ***P < 0.001 vs age-matched CTR; ◦*P* < 0.05, ^○○^*P* < 0.01 vs age-matched FD; +++*P* < 0.001 vs age-matched FD + PC1. Treatments: (A) 10 weeks: F (3,20) = 14.12, *P* < 0.0001; 25 weeks: F (3,20) = 8.328, *P* = 0.0009 (B) 10 weeks: F (3,20) = 0.8295, *P* = 0.4932; 25 weeks: F (3,20) = 5.160, *P* = 0.0084 (C) 10 weeks: F (3,20) = 2.140, *P* = 0.1271; 25 weeks: F (3,20) = 1.521, *P* = 0.2397 (D) 10 weeks: F (3,20) = 0.09864, *P* = 0.9599; 25 weeks: F (3,20)= 0.7582, *P* = 0.5307 (E) 10 weeks: F (3,20) = 7.811, *P* = 0.0012; 25 weeks: F (3,20) = 24.30, *P* < 0.0001 (F) 10 weeks: F (3,20) = 7.803, *P* = 0.0017; 25 weeks: F (3,20) = 2.568, *P* = 0.0832. IL, interleukin; PKR, prokineticin receptors; PKS, Prokineticin System; TNF-α, tumor necrosis factor-α.

The colon-rectum (Fig. 2) of both young and adult pathological mice was characterized by a clear upregulation of PK2 mRNA levels (panel A; *P* < 0.01) compared with the relative control groups, and treatment with both drugs reduced PK2 overexpression (*P* < 0.05). In the early stage of FD (10 weeks), the prokineticin receptors PKR1 (panel B) and PKR2 (panel C) did not undergo any significant modulations (*P* > 0.05), while as the disease progressed, the PKR1 receptor underwent overexpression (*P* < 0.05), which however was not significantly counteracted by the treatments.

Intestinal inflammation was also supported by the overexpression of some proinflammatory cytokines (panels D-F). In both young and adult pathological mice, IL-1β mRNA expression levels (panel E) were markedly increased (*P* < 0.05), while TNF-α ones (panel F) were altered only in an early condition of the disease (*P* < 0.05), but not significantly modulated in an advanced phase (*P* > 0.05). No alteration of IL-6 levels was ever observed (panel D). Both pharmacological interventions significantly reduced the inflammatory state (*P* < 0.05) in young mice. In the more advanced phase of FD, only PC1 counteracted the upregulation of IL-1β (*P* < 0.01 vs FD mice).

**Figure 3.**
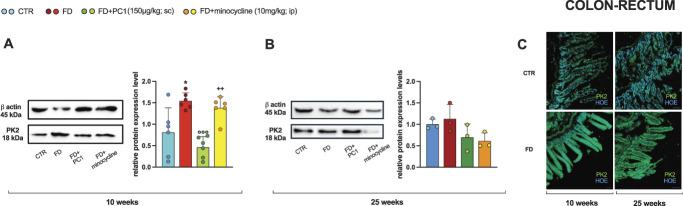
Colon-rectum: PK2 protein levels. Protein expression levels (western blot) of PK2 in (A) young (10-week-old) and (B) adult (25-week-old) mice were evaluated in the colon-rectum (after 14 days of pharmacological treatment). Results are normalized to the housekeeping protein β-actin and expressed as fold over the age-matched CTR group. Ten-week-old mice data are expressed as the mean ± SD of 6 animals/group; 25-week-old mice data are expressed as the mean ± SD of 3 animals/group. Statistical analyses were performed by 1-way ANOVA followed by the Šidák post hoc test. *P < 0.05 vs age-matched CTR; ^○○○^*P* < 0.001 vs age-matched FD; ++*P* < 0.01 vs age-matched FD + PC1. Treatments: (A) 10 weeks: F (3,20) = 12.28, *P* < 0.0001 (B) 25 weeks: F (3,8) =2.743, *P* = 0.1128. (C) Representative images of colon-rectum sections after IHC staining for PK2 in young (10-week-old) and adult (25-week-old) CTR and FD mice. Scale bars: 100 μm. ANOVA, analysis of variance; FD, Fabry–Anderson disease; IHC, immunohistochemistry; PK, prokineticin.

Colon-rectum PK2 levels were also evaluated as protein (Fig. 3). Western blot analyses confirmed the chemokine overexpression in young pathological mice compared with their controls (panel A; *P* < 0.05). In addition, the positive effect of PC1 was confirmed (*P* < 0.001), but not the efficacy of minocycline (*P* > 0.05). In the presence of more advanced stages of the disease (panel B), PK2 protein levels did not change and the 2 compounds, consequently, had no effect.

The overexpression of PK2 protein levels was also validated with qualitative immunohistochemistry (IHC) evaluations (panel C) that confirmed quantitative data, showing a stronger fluorescent intensity of the PK2 signal (green) in the gut of both 10-and 25-week-old pathological mice compared with their age-matched controls.

### 3.3. Sciatic nerve: prokineticin system and neuroinflammatory markers and effects of PC1 and minocycline treatments

**Figure 4.**
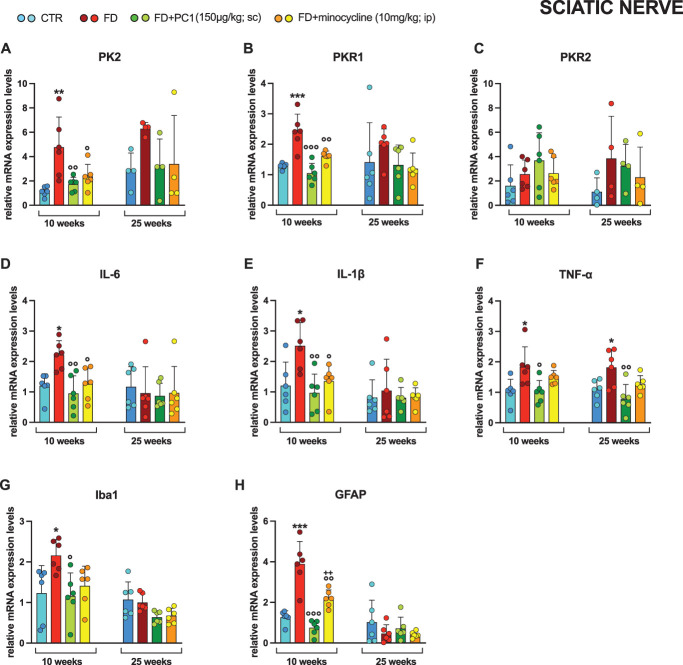
Sciatic nerve: mRNA levels of PKS and neuroinflammatory markers and effects of PC1 and minocycline treatments. mRNA expression levels (RT-qPCR) of the PKS members (A) PK2, (B) PKR1 and (C) PKR2, proinflammatory cytokines (D) IL-6, (E) IL-1β, and (F) TNF-α, (G) macrophage marker Iba1 and (H) Schwann cell marker GFAP were evaluated in the sciatic nerve (after 14 days of pharmacological treatment). Results are normalized to the housekeeping gene GAPDH and expressed as fold over the age-matched CTR group. Data are the mean ± SD of 4 to 6 animals/group. Statistical analyses were performed by one-way ANOVA followed by the Šidák post hoc test. *P < 0.05, **P < 0.01, ***P < 0.001 vs age-matched CTR; ◦*P* < 0.05, ^○○^*P* < 0.01, ^○○○^*P* < 0.001 vs age-matched FD; ++*P* < 0.01 vs age-matched FD + PC1. Treatments: (A) 10 weeks: F (3,20) = 7.912, *P* = 0.0011; 25 weeks: F (3,12) = 1.745, *P* = 0.2110 (B) 10 weeks: F (3,20) = 18.91, *P* < 0.0001; 25 weeks: F (3,20) = 1.077, *P* = 0.3814 (C) 10 weeks: F (3,20) = 1.559, *P* = 0.2305; 25 weeks: F (3,12) = 1.005, *P* = 0.4243 (D) 10 weeks: F (3,20) = 7.587, *P* = 0.0014; 25 weeks: F (3,20) = 0.1821, *P* = 0.9073 (E) 10 weeks: F (3,20) = 6.204, *P* = 0.0037; 25 weeks: F (3,20) = 0.1870, *P* = 0.9040 (F) 10 weeks: F (3,20) = 4.867, *P* = 0.0106; 25 weeks: F (3,20) = 6.058, *P* = 0.0042 (G) 10 weeks: F (3,20) = 4.306, *P* = 0.0169; 25 weeks: F (3,20) = 4.075, *P* = 0.0207 (H) 10 weeks: F (3,20) = 27.75, *P* < 0.0001; 25 weeks: F (3,20) = 1.119, *P* = 0.3648. ANOVA, analysis of variance; FD, Fabry–Anderson disease; IL, interleukin; PKR, prokineticin receptors; PKS, Prokineticin System; TNF-α, tumor necrosis factor-α.

A clear neuroinflammatory state was found in the sciatic nerve (Fig. 4) of young FD mice. Indeed, high mRNA levels of PK2 (panel A, *P* < 0.01) and PKR1 (panel B, *P* < 0.001), but not of PKR2 (panel C, *P* > 0.05), were observed. Both PC1 and minocycline significantly reduced these alterations (*P* < 0.05); prokineticin system alterations were less evident in 25-week-old mice.

In support of this initial PK system upregulation, 10-week-old mice were also characterized by increased levels of proinflammatory cytokines (IL-6, IL-1β, and TNF-α; panels D-F, *P* < 0.05), which were fully counteracted by PC1, showing a clear and extensive anti-inflammatory effect (*P* < 0.05). Conversely, minocycline reduced IL-6 and IL-1β (*P* < 0.05), but not TNF-α (*P* > 0.05) overexpression. Once again, neuroinflammation at this level seems to subside over time because only higher levels of TNF-α (*P* < 0.05) were observed in adult mice, rebalanced by treatment with PC1 (*P* < 0.01) but not with minocycline (*P* > 0.05).

Besides, the macrophage marker Iba1 (panel G) and the Schwann cell marker GFAP (panel H) levels highlight the different neuroinflammatory states existing between earlier and more advanced phases of FD. Indeed, only in young FD mice, but not in 25-week-old animals, a significant increase in both expression levels (*P* < 0.05) was observed. PC1 treatment was effective in counteracting both Iba1 and GFAP overexpression (*P* < 0.05), while minocycline only controlled GFAP high levels (*P* < 0.01) but less effectively than the antagonist of prokineticin receptors (*P* < 0.01).

### 3.4. Dorsal root ganglia: levels of prokineticin system, neuroinflammatory markers, epigenetic regulators, and effects of PC1 and minocycline treatments

**Figure 5.**
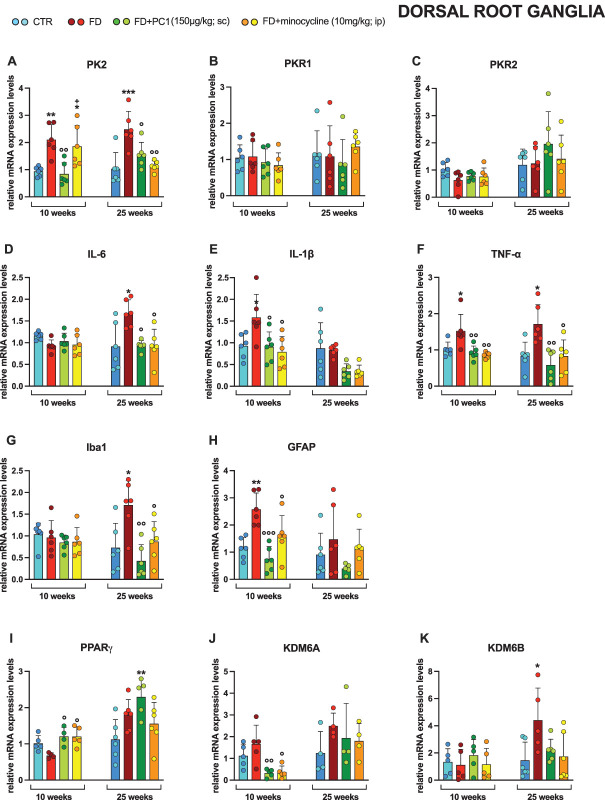
Dorsal root ganglia: mRNA levels of PKS, neuroinflammatory, epigenetic regulators and effects of PC1 and minocycline treatments. mRNA expression levels (RT-qPCR) of the PKS members (A) PK2, (B) PKR1 and (C) PKR2, proinflammatory cytokines (D) IL-6, (E) IL-1β, and (F) TNF-α, (G) macrophages marker Iba1, (H) satellite glial cell marker GFAP, (I) PPARγ, (J) KDM6A and (K) KDM6B were evaluated in the dorsal root ganglia (after 14 days of pharmacological treatment). Results are normalized to the housekeeping gene GAPDH and expressed as fold over the age-matched CTR group. Data are (A–H) the mean ± SD of 4 to 6 animals/group. Statistical analyses were performed by 1-way ANOVA followed by the Šidák post hoc test. *P < 0.05, **P < 0.01, ***P < 0.001 vs age-matched CTR; ◦*P* < 0.05, ^○○^*P* < 0.01, ^○○○^*P* < 0.001 vs age-matched FD; +*P* < 0.05 vs age-matched FD + PC1. Treatments: (A) 10 weeks: F (3,20) = 8.931, *P* = 0.0006; 25 weeks: F (3,20) = 10.22, *P* = 0.0003 (B) 10 weeks: F (3,20) = 0.5349, *P* = 0.6636; 25 weeks: F (3,20) = 0.5671, *P* = 0.6430 (C) 10 weeks: F (3,20) = 2.232, *P* = 0.1158; 25 weeks: F (3,20) = 1.015, *P* = 0.4069 (D) 10 weeks: F (3,20) = 2.239, *P* = 0.1150; 25 weeks: F (3,20) = 6.236, *P* = 0.0037 (E) 10 weeks: F (3,20) = 5.132, *P* = 0.0086; 25 weeks: F (3,20) = 4.947, *P* = 0.0099 (F) 10 weeks: F (3,20) = 7.602, *P* = 0.0014; 25 weeks: F (3,20) = 7.381, *P* = 0.0016 (G) 10 weeks: F (3,20) = 0.5217, *P* = 0.6723; 25 weeks: F (3,20) = 7.345, *P* = 0.0017 (H) 10 weeks: F (3,20) = 11.61, *P* = 0.0001; 25 weeks: F (3,20) = 1.768, *P* = 0.1856 (I) 10 weeks: F (3,14) = 5.393, *P* = 0.0112; 25 weeks: F (3,19) = 4.826, *P* = 0.0116 (J) 10 weeks: F (3,16) = 7.005, *P* = 0.0032; 25 weeks: F (3,12) = 0.9353, *P* = 0.4538 (K) 10 weeks: F (3,16) = 0.4278, *P* = 0.7358; 25 weeks: F (3,19) = 3.613, *P* = 0.322. FD, Fabry–Anderson disease; IL, interleukin; PKR, prokineticin receptors; PKS, Prokineticin System; TNF-α, tumor necrosis factor-α.

Considering the PKS, in the DRG (Fig. 5), only PK2 levels (panel A) increased significantly in both young and adult FD mice, compared with the relative controls (*P* < 0.01). Furthermore, the treatment with PC1 brought the expression levels of PK2 back to control values in both groups (*P* < 0.05). Minocycline was effective only in the advanced phase of the disease (*P* < 0.01), while it had no positive effect in young mice, at variance from PC1 (*P* < 0.05). PKR1 (panel B) and PKR2 (panel C) did not undergo any modulation either in young or adult FD mice (*P* > 0.05). Regarding proinflammatory cytokines, slightly different modulations were observed depending on the stage of the pathology. IL-6 mRNA levels (panel D) were upregulated only in an advanced stage of the disease (*P* < 0.05) and both treatments counteracted this overexpression (*P* < 0.05). On the contrary, IL-1β (panel E) was overexpressed only in FD young animals (*P* < 0.05). Both pharmacological treatments reduced the expression levels to basal values (*P* < 0.05) while TNF-α (panel F) was significantly upregulated both in the early and in the advanced stages of the pathology compared with the relative controls (*P* < 0.05), and again both PC1 and minocycline downregulated its expression (*P* < 0.05).

Besides, markers of glial and immune cells showed opposite modulations in the 2 analyzed age groups. In young FD mice, indeed, no variation in the expression levels of the macrophage marker Iba1 (panel G) was recorded (*P* > 0.05), but the same marker was significantly upregulated in the more advanced phase of the disease compared with the relative control mice (*P* < 0.05). Both drug treatments downregulated Iba1 expression in adulthood (*P* < 0.05). On the contrary, a significant increase in the expression levels of the satellite glial cell marker GFAP (panel H) was observed only in young FD mice compared with their corresponding controls (*P* < 0.01). Treatment with PC1 significantly reduced these values (*P* < 0.001 vs FD mice) more effectively than minocycline (*P* < 0.05 vs FD mice).

Interestingly, both PC1 and minocycline treatments increased PPARγ gene expression in young FD mice (panel I, *P* < 0.05 vs FD mice). However, a significant upregulation of this nuclear receptor was observed in PC1-treated FD adult mice only regarding CTRs (*P* < 0.01). Data also showed that PC1 and minocycline decreased KDM6A mRNA levels (panel J) at the early stage of FD (*P* < 0.05 vs FD mice). It is worth noting that KDM6B gene expression (panel K) was significantly higher in FD adult mice than in CTR animals (*P* < 0.05) whereas PC1- or minocycline-treated FD mice did not show significant differences in KDM6B mRNA levels compared with the age-matched CTR group (*P* > 0.05).

The overexpression of PK2 in DRG of both young and adult FD mice was confirmed by IHC experiments, as reported in Supplementary File 1, http://links.lww.com/PAIN/C393. Representative images showed an increase in the fluorescent intensity of the PK2 signal (green) in the DRG of both young and adult FD mice, compared with their age-matched controls. Although we did not perform colocalization of PK2 with specific markers of the different DRG cell populations, we can presume that PK2 is expressed mainly in neurons based on the distribution of its immunostaining.

**Figure 6.**
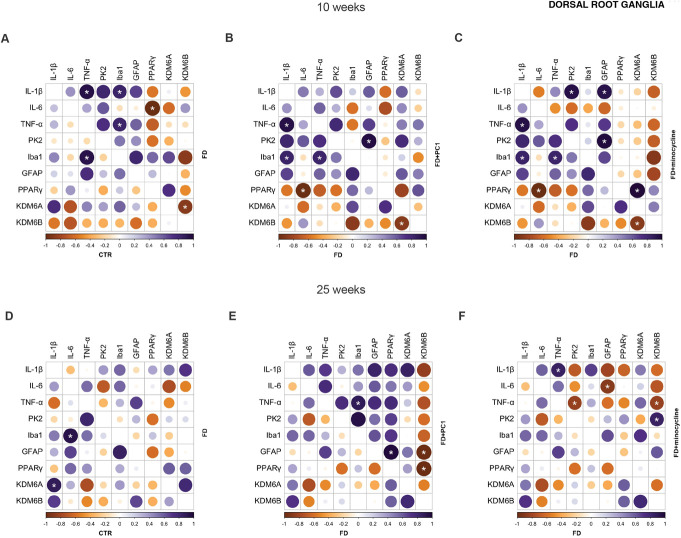
Dorsal root ganglia: correlation matrix showing the relationship between neuroinflammatory genes. (A–C) 10-week-old mice; (D–F) 25-week-old mice. (A and D) CTR: bottom-left; FD: top-right. (B and E) FD: bottom-left; FD + PC1: top-right. (C and F) FD: bottom-left; FD + minocycline: top-right. Pearson correlation coefficients have been computed. The plot shows the *P*-value for each pair of variables (n = 5-6 mice/group). The level of significance was set at *P* < 0.05 (*). Correlation ranges from −1 to +1. Positive values (violet) and negative values (orange) suggest positive and negative correlations, respectively. Color intensity and the size of the circle are indicative of the strength of the correlation. FD, Fabry–Anderson disease.

We constructed Pearson correlation matrix for the DRG (Fig. 6) of young and adult mice to investigate the relationship between the expression of genes involved in neuroinflammatory processes within each group. The FD condition determined changes in the neuroinflammatory network at both the early and late stages of the disease as compared with the CTR group (panels A, D). Interestingly, PC1 or minocycline treatment differently modulated the correlation pattern among the investigated genes in young and adult FD mice (panels B, C, E, F). Values of each Pearson correlation coefficient and *P*-value are summarized in Tables A1-A6 (see Supplementary files, http://links.lww.com/PAIN/C393).

### 3.5. Spinal cord: prokineticin system and neuroinflammatory markers and effects of PC1 and minocycline treatments

**Figure 7.**
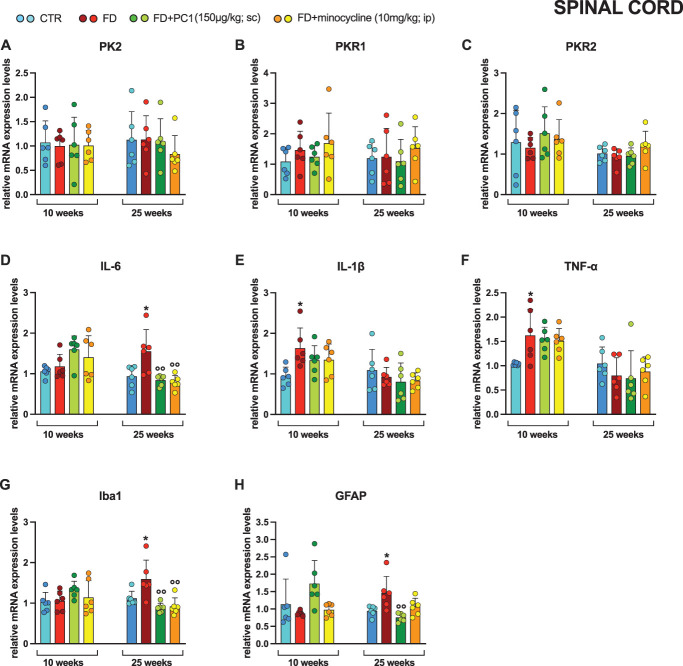
Spinal cord: mRNA levels of PKS and neuroinflammatory markers and effects of PC1 and minocycline. mRNA expression levels (RT-qPCR) of the PKS members (A) PK2, (B) PKR1 and (C) PKR2, proinflammatory cytokines (D) IL-6, (E) IL-1β, and (F) TNF-α, (G) microglia marker Iba1 and (H) astrocytes marker GFAP were evaluated in the spinal cord (after 14 days of pharmacological treatment). Results are normalized to the housekeeping gene GAPDH and expressed as fold over the age-matched CTR group. Data are the mean ± SD of 6 animals/group. Statistical analyses were performed by 1-way ANOVA followed by the Šidák post hoc test. *P < 0.05 vs age-matched CTR; ^○○^*P* < 0.01 vs age-matched FD. Treatments: (A) 10 weeks: F (3,20) = 0.3411, *P* = 0.9913; 25 weeks: F (3,20) = 0.5229, *P* = 0.6715 (B) 10 weeks: F (3,20) = 0.9708, *P* = 0.4260; 25 weeks: F (3,20) = 0.3878, *P* = 0.7630 (C) 10 weeks: F (3,20) = 0.4009, *P* = 0.7539; 25 weeks: F (3,20) = 1.428, *P* = 0.2642 (D) 10 weeks: F (3,20) = 2.787, *P* = 0.0672; 25 weeks: F (3,20) = 7.518, *P* = 0.0015 (E) 10 weeks: F (3,20) = 3.406, *P* = 0.0376; 25 weeks: F (3,20) = 0.6931, *P* = 0.5670 (F) 10 weeks: F (3,20) = 3.981, *P* = 0.0224; 25 weeks: F (3,20) = 0.6343, *P* = 0.6016 (G) 10 weeks: F (3,20) = 1.136, *P* = 0.2824; 25 weeks: F (3,20) = 8.352, *P* = 0.0008 (H) 10 weeks: F (3,20) = 3.490, *P* = 0.0348; 25 weeks: F (3,20) = 6.563, *P* = 0.0029. ANOVA, analysis of variance; Iba1, ionized calcium-binding adapter molecule 1; IL, interleukin; PKR, prokineticin receptors; PKS, Prokineticin System; TNF-α, tumor necrosis factor-α.

In the spinal cord (Fig. 7), PK2 (panel A), PKR1 (panel B), and PKR2 (panel C) expression was not altered either in young (10-week-old) or in adult (25-week-old) mice in any experimental condition (*P* > 0.05). No alterations in IL-6 mRNA levels (panel D) were observed in the spinal cord of young mice (*P* > 0.05); however, IL-6 was upregulated in adult FD mice compared with the respective control animals (*P* < 0.05) and both PC1 and minocycline were able to significantly lower its expression level back to CTR values (*P* < 0.01).

Only in young FD mice, a significant increase in expression levels of IL-1β (panel E, *P* < 0.05) and TNF-α (panel F, *P* < 0.05) was observed compared with their CTR mice. Neither of the 2 pharmacological treatments significantly counteracted the upregulation of these 2 proinflammatory cytokines. Finally, no variation in either microglial Iba1 (panel G) or astrocyte GFAP (panel H) marker expression levels was observed in young mice (*P* > 0.05). However, both markers were significantly overexpressed in adult FD mice compared with age-matched CTR animals (*P* < 0.05), and both PC1 and minocycline significantly decreased Iba1 overexpression (*P* < 0.01). Meanwhile, GFAP gene expression increased in adult FD mice was significantly counteracted only by PC1 treatment (*P* < 0.01).

**Figure 8.**
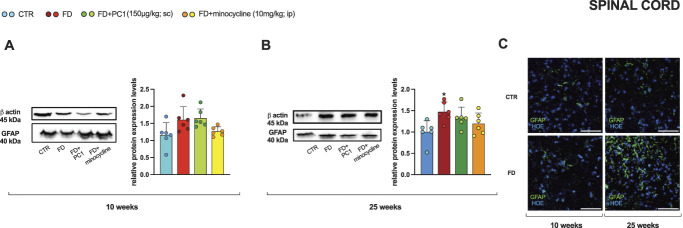
Spinal cord: GFAP protein levels, effects of PC1 and minocycline and qualitative immunohistochemistry. Protein expression levels (Western Blot) of the astrocyte marker GFAP (A) in young (10-week-old) and (B) adult (25-week-old) mice were evaluated in the spinal cord (after 14 days of pharmacological treatment). Results are normalized to the housekeeping protein β-actin and expressed as fold over the age-matched CTR group. Data are the mean ± SD of 6 animals/group. Statistical analyses were performed by 1-way ANOVA followed by the Šidák post hoc test. *P < 0.05 vs age-matched CTR. Treatments: (A) 10 weeks: F (3,20) = 3.956, *P* = 0.0229 (B) 25 weeks: F (3,20) = 3.955, *P* = 0.0230. (C) Immunohistochemistry qualitative images of GFAP signal in the spinal cord are provided. Scale bars: 50 μm. ANOVA, analysis of variance.

The time-dependent modulation of the mRNA levels of astrocytic marker GFAP in the spinal cord was also confirmed at the protein level (Fig. 8). Western blot analysis revealed the absence of modulation in young pathological mice (panel A) and confirmed increased protein levels in the advanced-stage disease (panel B, *P* < 0.05). However, in this context, the positive effect of drugs was less evident. Moreover, a qualitative immunofluorescence evaluation (panel C) confirmed these data, showing reactive astrogliosis in the spinal cord of adult FD mice only. Representative IHC images showed differences in the density and morphology of GFAP+ cells (in green) with hypertrophic and more numerous cells, typical signs of astrogliosis, in adult FD mice compared with young FD mice and CTR mice of both ages.

## 4. Discussion

Neuropathic pain, resulting from globotriaosylceramide (Gb3) accumulation in the nervous system, is the main neurological symptom in FD, affecting more than 80% of patients from childhood.^11,61,63^ Current analgesics and innovative disease-specific therapies, such as ERT or chaperone therapy, are not effective in fully controlling FD-associated pain.^26,29,52,55,62^

In this study, we provide compelling evidence that neuroinflammation plays a central role in the pathogenesis of FD-related NP and that its pharmacological suppression may offer an effective analgesic strategy. To control neuroinflammation, we tested 2 distinct approaches: minocycline, a microglial inhibitor,^22^ and PC1, a specific receptor antagonist of the PKS. This system is a novel family of chemokines, composed of the ligand PK2 and the G-protein-coupled receptors PKR1/PKR2.^6,19,21^ It is widely expressed in neurons, immune, and glial cells and is known to be upregulated in both inflammatory and NP states.^3,6,15,21,37,41,42,45^

Consistent with prior findings,^32,59^ the absence of the *GLA* gene induced, already at 10 weeks of age, both mechanical allodynia and thermal hyperalgesia that persisted as the disease progressed. Interestingly, abdominal pain and cold allodynia showed differences across disease stages. Young FD mice showed severe abdominal pain, which, in contrast, was attenuated in adult mice. This observation is quite new and consistent with a recent work^18^ and clinical observations. Indeed, it aligns with gastrointestinal symptoms observed in FD children, likely due to autonomic nervous system impairment, assumingly connected to enteric malfunction of neurons, while adult patients with FD report abdominal pain less frequently.^9,17,26,66^

Moreover, we noted changes in cold sensitivity over time, as also reported in the literature.^27,32,60^ These modifications may reflect reduced intraepidermal nerve fiber density or downregulation of cold-sensitive channels such as TRPM8, which plays a physiological role in detecting low temperatures.^27^

Both minocycline and PC1 treatments significantly alleviated sensory abnormalities in acute, with a slightly higher efficacy of minocycline. This may depend on their distinct mechanisms of action: While both drugs inhibit the production and release of proinflammatory mediators and immune recruitment,^6,12,20,30^ PC1 could act in this sense only secondary to the blockade of the PKS. Furthermore, minocycline additionally targets phospholipase A2 and COX2,^12^ and both enzymes are not modulated by PK2 or PKS antagonist PC1.^46^ Nevertheless, chronic treatment with either agent similarly and successfully reduced pain in FD animals at both early and advanced disease stages, with effects sustained over time, after just 1 week.

In parallel with the behavioral data related to abdominal pain, we observed marked colon-rectal inflammation in FD mice of both ages, with elevated PK2, PKR1, and proinflammatory cytokines. These results are consistent with the known high intestinal expression and upregulation of PKS in chronic inflammation.^4,5^

To assess the presence of neuroinflammation, we measured the levels of 3 key proinflammatory cytokines: IL-6, IL-1β, and TNF-α. Given the crucial role of glial cells, we also investigated the involvement of microglia/macrophages, satellite glial cells, and astrocytes by assessing the most widely used markers of their activation (Iba1 and GFAP). Moreover, we also studied PPARγ, for its recently recognized role in reducing microglia activation and contrasting neuroinflammation.^10,14,36^

We performed such analyses in the peripheral nerve fibers, known for being particularly damaged in patients with FD,^63^ in DRG, indicated by several studies as the most modified pain stations^16,31^ and, for the first time, also in the spinal cord.

Our biochemical results relating to the the peripheral nervous system (PNS) highlight that sciatic nerves of young FD mice exhibited early and robust neuroinflammation, characterized by elevated proinflammatory cytokines and activated Schwann cells (as shown by GFAP overexpression) and macrophages (demonstrated by Iba1 upregulation). Moreover, for the first time, we also detected high levels of the chemokine PK2 and of its receptor PKR1, but not of PKR2, which is poorly expressed in the PNS.^4^ These alterations diminished with age.

In both FD stages, DRG were characterized by cytokines and PK2 upregulation and, initially, by a clear activation of satellite glial cells linked to high levels of GFAP, followed by a remarkable upregulation of the macrophage marker Iba1 along with the disease progression. Presumably, Schwann and satellite glial cells were damaged and activated in response to the accumulation of Gb3; these events attracted in situ infiltrating immune cells, which migrated from the sciatic nerve toward the DRG.^65^

Taken together, these data show for the first time a precocious alteration of the PNS of FD mice, which involves non-neuronal cells and PK2 upregulation, thus reinforcing their contribution to FD-associated NP.^4,6,15,20,21,39,46^

In the central nervous system, in an early phase of the disease, the spinal cord showed mild neuroinflammation with elevated cytokine levels only, while pronounced microgliosis (elevated Iba1 levels) and astrogliosis (elevated GFAP levels) developed over time. This delayed central neuroinflammation suggests a peripheral-to-central progression of neuroinflammatory processes and supports the hypothesis that persistent nociceptive input from the PNS drives central sensitization. This evidence is innovative because, to date, alterations of the spinal cord related to pain in FD mice have not been consistently reported. Furthermore, this novel observation may explain the limited efficacy of ERT in controlling pain, as established neuroplastic changes in the spinal cord are difficult to reverse.^29,55^

Both chronic PC1 and minocycline treatments reduced peripheral (sciatic nerve, DRG) and central (spinal cord) neuroinflammation, and gut inflammation, by inhibiting proinflammatory cytokines production and reducing immune/glial cell activation. However, PC1 had a broader impact on PK2/PKR1 modulation and astrocytic activity, particularly in the DRG and spinal cord. It remains possible that longer minocycline treatment could achieve similar PKS modulation.

Notably, together with the downregulation of PK2, IL-1β, and TNF-α, PC1 also increased PPARγ expression in DRG of animals of both ages. This evidence suggests that its anti-inflammatory effects may be mediated by both suppression of proinflammatory pathways and activation of endogenous anti-inflammatory mechanisms.^10,14,28,36,48^

Moreover, in DRG at later disease stages, we also detected increased expression of KDM6B, an epigenetic regulator, together with IL-6 overexpression. This supports recent work linking KDM6B-mediated H3K27me3 demethylation to the binding of transcription activators (ie, STAT3) to the IL-6 promoter, driving IL-6 overexpression in the spinal cord dorsal horns and DRG in NP conditions.^34,35,51,53^

PC1 and minocycline normalized these alterations, further confirming their potential to counteract persistent neuroinflammatory pathways in FD. Correlation analysis further strengthens these conclusions by showing how neuroinflammatory patterns are altered in treated animals. Although both drugs produced similar behavioral changes, they modulated neuroimmune signaling in a different and age-dependent manner, consistent with their unique pharmacodynamic profiles.

Overall, our findings highlight that PKS and neuroinflammation in both the PNS and central nervous system contribute to FD-associated pain. Pharmacological strategies that attenuate PKS signaling, glial activation, and proinflammatory cytokine release, while enhancing anti-inflammatory pathways, can provide substantial analgesic benefits, even with different mechanisms of action.

One limitation of the study may be the use of male mice only. However, FD is an X-linked disease and, overall, female patients with FD show a milder phenotype, later onset of symptoms, and slower disease progression.^38^ In addition, GLA^*−/−*^ male mice consistently recapitulate the classical FD phenotype (total enzyme absence) manifested only by male patients,^13^ whereas female GLA^−/−^ mice do not represent the clinical situation in women. Moreover, in the heterozygous female mice, the X inactivation process might impair the reproducibility of results.^18^ However, future studies including female mice are warranted to assess sex-dependent differences and better reflect the patient population and translate to clinics.

For a translational perspective, it is important to highlight that PC1 represents a promising candidate because no adverse effects have been reported in extensive preclinical models both in vitro and in vivo*,*^6,15,21,39,43^ and its specificity for PKS makes it particularly attractive for targeted pain therapy in FD. In addition, the study acquires further value considering drug-repurposing potential for minocycline, which, although already used for some pain syndromes, is not currently prescribed for FD-related symptoms.

## Conflict of interest statement

The authors have no conflicts of interest to declare.

## Supplementary Material

## Appendix A. Supplemental digital content

Supplemental digital content associated with this article can be found online at http://links.lww.com/PAIN/C393.
