# Supplementary material for: The prokineticin system and glia cells as pharmacological targets to control neuroinflammation and to relieve pain in a murine model of Fabry–Anderson disease
Source: Pain. 2025 Sep 25;167(2):428–42. doi: 10.1097/j.pain.0000000000003818 (PMC12794355; doi:10.1097/j.pain.0000000000003818)
Supplement: Supplementary file 1 [file jop-167-428-s001.pdf]

Supplementary Figure 1

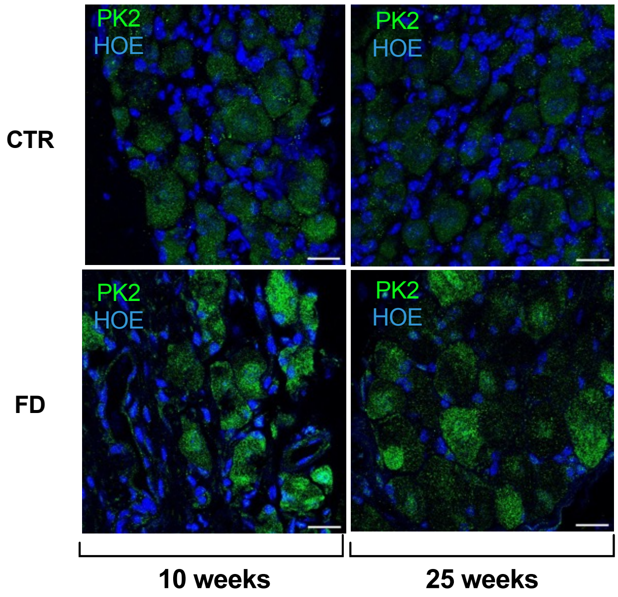

Supplementary Figure 1| Dorsal root ganglia: qualitative IHC staining of PK2 expression.
(top left) PK2 protein expression in 10-week-old control mice; (bottom left) PK2 protein expression in 10-week-old FD mice; (top right) PK2 protein expression in 25-week-old control mice; (bottom right) PK2 protein expression in 25-week-old FD mice. Scale bars: 20 µm.

Supplementary Table A1

	IL-1β		IL-6		TNF-α		PK2		Iba1		GFAP		PPARγ		KDM6A		KDM6B	
	r	p	r	p	r	p	r	p	r	p	r	p	r	p	r	p	r	p
IL-1β	1	NA	0.412	0.417	0.944	0.00465	0.811	0.0502	0.831	0.0404	0.668	0.147	-0.565	0.435	0.063	0.92	-0.464	0.432
IL-6	0.25	0.633	1	NA	0.359	0.484	0.61	0.199	-0.154	0.771	-0.083	0.876	-0.996	0.00444	-0.61	0.275	0.41	0.493
TNF-α	0.297	0.567	-0.162	0.758	1	NA	0.745	0.089	0.826	0.0429	0.577	0.231	-0.676	0.324	-0.153	0.806	-0.16	0.798
PK2	-0.122	0.818	-0.11	0.836	-0.183	0.728	1	NA	0.503	0.309	0.433	0.391	-0.583	0.417	-0.362	0.55	-0.028	0.964
Iba1	0.453	0.366	-0.195	0.712	0.938	0.0057	-0.23	0.662	1	NA	0.806	0.0526	0.554	0.446	0.642	0.243	-0.874	0.0528
GFAP	0.193	0.715	0.123	0.816	0.754	0.0834	0.258	0.622	0.537	0.272	1	NA	0.277	0.723	0.016	0.979	-0.36	0.552
PPARγ	-0.231	0.709	0.407	0.497	0.075	0.904	-0.384	0.523	0.228	0.712	-0.277	0.652	1	NA	0.737	0.263	-0.492	0.508
KDM6A	0.758	0.137	-0.725	0.165	0.464	0.431	0.379	0.529	0.576	0.31	0.261	0.671	-0.382	0.526	1	NA	-0.883	0.0471
KDM6B	-0.56	0.326	-0.714	0.175	-0.362	0.55	-0.387	0.52	-0.344	0.571	-0.602	0.283	-0.372	0.537	0.055	0.93	1	NA

Supplementary Table A1| Correlation matrix between neuroinflammatory genes in the DRGs of 10-week-old mice.
The table presents Pearson's correlation coefficients (r). The top-right portion of the table shows the correlation values for the FD group, while the bottom-left portion displays the corresponding values for the CTR group. p-values (p) are included to indicate the statistical significance of the correlations.

Supplementary Table A2

	IL-1β		IL-6		TNF-α		PK2		Iba1		GFAP		PPARγ		KDM6A		KDM6B	
	r	p	r	p	r	p	r	p	r	p	r	p	r	p	r	p	r	p
IL-1β	1	NA	0.492	0.321	0.307	0.554	0.634	0.176	0.212	0.687	0.593	0.215	-0.607	0.278	0.6	0.285	-0.153	0.806
IL-6	0.412	0.417	1	NA	0.577	0.231	0.312	0.547	-0.589	0.219	0.131	0.805	-0.753	0.141	0.444	0.454	-0.23	0.71
TNF-α	0.944	0.00465	0.359	0.484	1	NA	0.786	0.0641	-0.569	0.238	0.674	0.142	-0.08	0.899	0.483	0.41	0.511	0.378
PK2	0.811	0.0502	0.61	0.199	0.745	0.089	1	NA	-0.034	0.949	0.961	0.00228	-0.277	0.652	0.766	0.131	0.302	0.621
Iba1	0.831	0.0404	-0.154	0.771	0.826	0.0429	0.503	0.309	1	NA	0.012	0.982	-0.112	0.858	0.194	0.755	-0.496	0.396
GFAP	0.668	0.147	-0.083	0.876	0.577	0.231	0.433	0.391	0.806	0.0526	1	NA	-0.097	0.877	0.605	0.28	0.481	0.412
PPARγ	-0.565	0.435	-0.996	0.00444	-0.676	0.324	-0.583	0.417	0.554	0.446	0.277	0.723	1	NA	-0.811	0.0958	0.624	0.261
KDM6A	0.063	0.92	-0.61	0.275	-0.153	0.806	-0.362	0.55	0.642	0.243	0.016	0.979	0.737	0.263	1	NA	-0.298	0.626
KDM6B	-0.464	0.432	0.41	0.493	-0.16	0.798	-0.028	0.964	-0.874	0.0528	-0.36	0.552	-0.492	0.508	-0.883	0.0471	1	NA

Supplementary Table A2| Correlation matrix showing the relationship between neuroinflammatory genes in the DRGs of 10-week-old mice

The table presents Pearson's correlation coefficients (r). The top-right portion of the table shows the correlation values for the FD+PC1 group, while the bottom-left portion displays the corresponding values for the FD group. p-values (p) are included to indicate the statistical significance of the correlations.

Supplementary Table A3

	IL-1β		IL-6		TNF-α		PK2		Iba1		GFAP		PPARγ		KDM6A		KDM6B	
	r	p	r	p	r	p	r	p	r	p	r	p	r	p	r	p	r	p
IL-1β	1	NA	-0.545	0.263	0.45	0.371	0.942	0.0049	0.155	0.769	0.935	0.0197	-0.111	0.889	-0.215	0.785	-0.664	0.336
IL-6	0.412	0.417	1	NA	-0.485	0.33	-0.606	0.203	-0.401	0.431	-0.638	0.247	-0.083	0.917	0.021	0.979	-0.07	0.93
TNF-α	0.944	0.00465	0.359	0.484	1	NA	0.721	0.106	0.106	0.841	0.699	0.189	-0.196	0.804	-0.301	0.699	-0.635	0.365
PK2	0.811	0.0502	0.61	0.199	0.745	0.089	1	NA	0.191	0.717	0.99	0.00122	-0.154	0.846	-0.257	0.743	-0.67	0.33
Iba1	0.831	0.0404	-0.154	0.771	0.826	0.0429	0.503	0.309	1	NA	0.656	0.229	-0.035	0.965	-0.06	0.94	-0.897	0.103
GFAP	0.668	0.147	-0.083	0.876	0.577	0.231	0.433	0.391	0.806	0.0526	1	NA	-0.192	0.808	-0.285	0.715	-0.792	0.208
PPARγ	-0.565	0.435	-0.996	0.00444	-0.676	0.324	-0.583	0.417	0.554	0.446	0.277	0.723	1	NA	0.993	0.0065	0.454	0.546
KDM6A	0.063	0.92	-0.61	0.275	-0.153	0.806	-0.362	0.55	0.642	0.243	0.016	0.979	0.737	0.263	1	NA	0.487	0.513
KDM6B	-0.464	0.432	0.41	0.493	-0.16	0.798	-0.028	0.964	-0.874	0.0528	-0.36	0.552	-0.492	0.508	-0.883	0.0471	1	NA

Supplementary Table A3. Correlation matrix showing the relationship between neuroinflammatory genes in the DRGs of 10-week-old mice.

The table presents Pearson's correlation coefficients (r). The top-right portion of the table shows the correlation values for the FD+ minocycline group, while the bottom-left portion displays the corresponding values for the FD group. p-values (p) are included to indicate the statistical significance of the correlations.

Supplementary Table A4

	IL-1β		IL-6		TNF-α		PK2		Iba1		GFAP		PPARγ		KDM6A		KDM6B	
	r	p	r	p	r	p	r	p	r	p	r	p	r	p	r	p	r	p
IL-1β	1	NA	-0.301	0.562	-0.035	0.947	0.4	0.432	0.569	0.239	-0.162	0.759	0.355	0.489	0.511	0.3	0.714	0.111
IL-6	0.395	0.439	1	NA	0.548	0.261	-0.698	0.123	0.562	0.246	-0.042	0.938	0.079	0.882	-0.751	0.0852	-0.52	0.29
TNF-α	-0.602	0.282	0.12	0.847	1	NA	-0.341	0.508	0.294	0.572	0.674	0.142	-0.044	0.934	-0.495	0.318	0.068	0.898
PK2	-0.194	0.712	-0.098	0.853	0.816	0.0923	1	NA	-0.156	0.768	0.288	0.579	-0.594	0.214	0.191	0.717	0.156	0.768
Iba1	0.492	0.322	0.939	0.0054	0.531	0.357	0.156	0.767	1	NA	-0.2	0.703	0.247	0.637	-0.248	0.635	0.005	0.993
GFAP	0.31	0.611	0.662	0.224	0.064	0.919	-0.22	0.722	0.854	0.0654	1	NA	-0.607	0.202	-0.395	0.439	-0.032	0.952
PPARγ	-0.281	0.59	0.55	0.258	0.426	0.474	-0.005	0.992	0.339	0.511	0.077	0.902	1	NA	0.596	0.212	0.582	0.225
KDM6A	0.884	0.0195	0.314	0.544	-0.757	0.138	-0.192	0.715	0.398	0.435	-0.22	0.722	-0.349	0.498	1	NA	0.797	0.0579
KDM6B	0.686	0.132	-0.108	0.839	-0.505	0.385	-0.383	0.453	-0.074	0.889	0.659	0.227	-0.377	0.461	0.378	0.46	1	NA

Supplementary Table A4. Correlation matrix showing the relationship between neuroinflammatory genes in the DRGs of 25-week-old mice.
The table presents Pearson's correlation coefficients (r). The top-right portion of the table shows the correlation values for the FD group, while the bottom-left portion displays the corresponding values for the CTR group. p-values (p) are included to indicate the statistical significance of the correlations.

Supplementary Table A5

	IL-1β		IL-6		TNF-α		PK2		Iba1		GFAP		PPARγ		KDM6A		KDM6B	
	r	p	r	p	r	p	r	p	r	p	r	p	r	p	r	p	r	p
IL-1β	1	NA	0.602	0.283	0.694	0.194	0.308	0.692	0.526	0.363	0.831	0.0816	0.753	0.142	0.847	0.0701	-0.847	0.0701
IL-6	-0.301	0.562	1	NA	0.766	0.131	-0.051	0.949	0.405	0.498	0.603	0.282	0.51	0.38	0.394	0.512	-0.481	0.412
TNF-α	-0.035	0.947	0.548	0.261	1	NA	0.759	0.241	0.896	0.0399	0.768	0.13	0.779	0.12	0.237	0.701	-0.732	0.16
PK2	0.4	0.432	-0.698	0.123	-0.341	0.508	1	NA	0.942	0.0577	0.535	0.465	0.674	0.326	-0.171	0.829	-0.61	0.39
Iba1	0.569	0.239	0.562	0.246	0.294	0.572	-0.156	0.768	1	NA	0.623	0.262	0.706	0.182	0.014	0.982	-0.652	0.234
GFAP	-0.162	0.759	-0.042	0.938	0.674	0.142	0.288	0.579	-0.2	0.703	1	NA	0.982	0.00283	0.517	0.373	-0.985	0.00226
PPARγ	0.355	0.489	0.079	0.882	-0.044	0.934	-0.594	0.214	0.247	0.637	-0.607	0.202	1	NA	0.377	0.532	-0.981	0.00319
KDM6A	0.511	0.3	-0.751	0.0852	-0.495	0.318	0.191	0.717	-0.248	0.635	-0.395	0.439	0.596	0.212	1	NA	-0.537	0.351
KDM6B	0.714	0.111	-0.52	0.29	0.068	0.898	0.156	0.768	0.005	0.993	-0.032	0.952	0.582	0.225	0.797	0.0579	1	NA

Supplementary Table A5. Correlation matrix showing the relationship between neuroinflammatory genes in the DRGs of 25-week-old mice.
The table presents Pearson's correlation coefficients (r). The top-right portion of the table shows the correlation values for the FD+PC1 group, while the bottom-left portion displays the corresponding values for the FD group. p-values (p) are included to indicate the statistical significance of the correlations.

Supplementary Table A6

	IL-1β		IL-6		TNF-α		PK2		Iba1		GFAP		PPARγ		KDM6A		KDM6B	
	r	p	r	p	r	p	r	p	r	p	r	p	r	p	r	p	r	p
IL-1β	1	NA	0.586	0.222	0.852	0.0313	-0.69	0.129	0.674	0.212	-0.801	0.104	-0.652	0.161	0.5	0.313	-0.614	0.195
IL-6	-0.301	0.562	1	NA	0.535	0.274	-0.486	0.329	0.152	0.807	-0.88	0.0487	0.028	0.958	0.196	0.71	-0.72	0.107
TNF-α	-0.035	0.947	0.548	0.261	1	NA	-0.834	0.0388	0.324	0.595	-0.595	0.29	-0.516	0.294	0.557	0.251	-0.832	0.0398
PK2	0.4	0.432	-0.698	0.123	-0.341	0.508	1	NA	-0.171	0.783	0.273	0.657	0.415	0.413	-0.399	0.434	0.841	0.036
Iba1	0.569	0.239	0.562	0.246	0.294	0.572	-0.156	0.768	1	NA	0.638	0.362	-0.259	0.673	0.726	0.165	-0.161	0.796
GFAP	-0.162	0.759	-0.042	0.938	0.674	0.142	0.288	0.579	-0.2	0.703	1	NA	-0.284	0.644	0.053	0.933	0.609	0.275
PPARγ	0.355	0.489	0.079	0.882	-0.044	0.934	-0.594	0.214	0.247	0.637	-0.607	0.202	1	NA	0.082	0.877	0.018	0.973
KDM6A	0.511	0.3	-0.751	0.0852	-0.495	0.318	0.191	0.717	-0.248	0.635	-0.395	0.439	0.596	0.212	1	NA	-0.596	0.212
KDM6B	0.714	0.111	-0.52	0.29	0.068	0.898	0.156	0.768	0.005	0.993	-0.032	0.952	0.582	0.225	0.797	0.0579	1	NA

Supplementary Table A6| Correlation matrix showing the relationship between neuroinflammatory genes in the DRGs of 25-week-old mice.
The table presents Pearson's correlation coefficients (r). The top-right portion of the table shows the correlation values for the FD+ minocycline group, while the bottom-left portion displays the corresponding values for the FD group. p-values (p) are included to indicate the statistical significance of the correlations.
